# Trueperella Pyogenes—Strain Diversity and Occurrence in Dairy Herds

**DOI:** 10.3390/pathogens13070534

**Published:** 2024-06-24

**Authors:** Nicole Wente, Stefanie Leimbach, Svenja Woudstra, Volker Krömker

**Affiliations:** 1 Department of Bioprocess Engineering and Microbiology, University of Applied Sciences and Arts Hannover, D-30453 Hannover, Germany; 2 Steinbeis Research Center Milk Science, D-32278 Kirchlengern, Germany; 3 Department of Veterinary and Animal Sciences, Faculty of Health and Medical Sciences, University of Copenhagen, Grønnegårdsvej 2, 1870 Frederiksberg C, Denmark

**Keywords:** Trueperella pyogenes, environmental, contagious, strain variety, occurrence, diversity, RAPD PCR

## Abstract

*Trueperella (T.) pyogenes* is a mastitis-causing pathogen formerly known to cause severe clinical mastitis (CM), especially during the summer, leading to milk losses and low recovery rates. Unfortunately, its transmission behavior within herds is unclear. The diversity and occurrence of *T. pyogenes* were monitored to gain an initial insight into the infection transmission behavior of *T. pyogenes* in dairy herds and to lay a foundation for following targeted investigations. CM milk samples were collected from German herds, and one Swedish farm was sampled for isolates from subclinical mastitis. All in all, 151 *T. pyogenes* isolates from 16 herds were isolated, identified by MALDI TOF analysis and typed with RAPD PCR. Of these, 17 isolates originated from subclinical mastitis cases. We found that *T. pyogenes* mastitis occurred year-round, and clinical mastitis cases were caused by multiple strains (31 affected animals/28 strains). Instances of multiple cows being infected with the same *T. pyogenes* strain were rare and typically only involved a small number of animals at a time. However, if several quarters of a cow were affected, it was likely the same strain. Unlike clinical infections, subclinical *T. pyogenes* infections, in one investigated farm, harbored a dominant strain. Additionally, we found that *T. pyogenes* infections tended to persist and stay within a herd for a minimum of 7 months in the same or different cows.

## 1. Introduction

**Figure 1:**
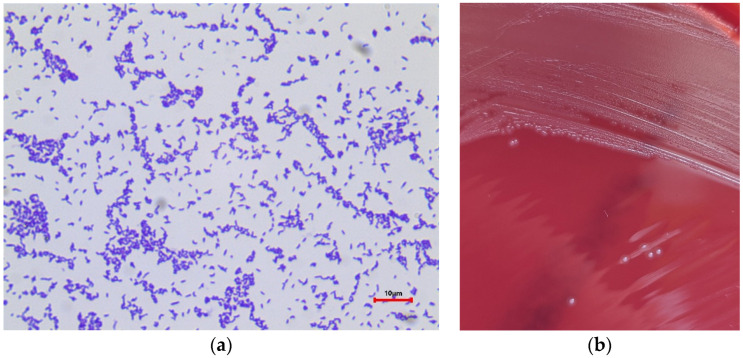
(**a**) Gram staining of *T. pyogenes*; (**b**) *T. pyogenes* colonies on esculin blood agar plate (Oxoid, Wesel, Germany) after incubation at 37 °C for 48 h.

Bovine mastitis is one of the most expensive and most frequently occurring diseases in dairy cows. This udder inflammation is usually caused by an infection of the mammary gland with pathogenic microorganisms. These microorganisms are categorized into two groups: environmental and contagious mastitis-causing pathogens. The source of environmental pathogen infections can be found in cows’ surroundings, intestinal tracts, or mucosal surfaces, whereas contagious microorganisms are mainly transmitted from cow to cow, e.g., during milking. *Trueperella* (*T*.) *pyogenes* (formerly *Arcanobacterium pyogenes*, *Actinomyces pyogenes*, and *Corynebacterium pyogenes*) [1] is a mastitis-causing pathogen that tends to be environmentally related. Nevertheless, it cannot yet be clearly assigned to one of these groups as the reservoirs and transmission routes of these bacteria are not understood to date. *T. pyogenes* is a Gram-positive, rod-shaped bacterium (Figure 1a) which was previously isolated from cows affected with wound infections and abscesses [2]. It is known to cause clinical, phlegmonous, and recurrent mastitis whereby the infection can appear in both lactating and dry cows [3]. *T. pyogenes* mastitis is supposed to occur in the summer in pastured cattle; therefore, flies are suspected to be vectors in infection transmission [2]. Conversely, no significant differences in *T. pyogenes* incidence by month or seasonality could be shown by several studies [3,4]. This casts doubt on the previously assumed epidemiology of *T. pyogenes* and calls into question the effectiveness of the respective control measures.

The type of husbandry, stabled or pastured cattle, seems to have no effect on the *T. pyogenes* colonization of the teat tips, conjunctivas, or oral cavities of cows [5]. Therefore, the source of infection and the routes of transmission are still unclear to date. Furthermore, *T. pyogenes* is suspected of being a secondary colonizer [3] which synergizes with other pathogens in the course of infection [6].

*T. pyogenes* expresses numerous virulence factors, like tissue damage by hemolytic exotoxin pyolysin [7], TatD DNases, biofilm formation [8], and adherence to host cells and colonization trough neuraminidases, binding proteins, and the expression of fimbriae [9]. Tamai et al. (2022) [6] investigated the expression levels of genes encoding pyolysin, fimbriae, neuraminidase, and collagen-binding protein (plo, fimA, nanH, and cbpA) in response to co-culture with other pathogens in mice study models. The expression levels increased significantly when *T. pyogenes* was co-cultured with *Fusobacterium necrophorum* and *Escherichia coli,* respectively. In contrast, *Lactobacillus plantarum* suppressed the expressions of all virulence factor genes. Rogovskyy et al. (2018) [10] detected different *T. pyogenes* genotypes for caprine and ovine isolates compared to bovine isolates. One genotype (plo/nanH/nanP/fimA/fimC) was only found in caprine and ovine isolates, while the other genotype (plo/nanP/fimA/fimC/fimE) was solely present in the isolates of bovine origin [10]. Like the pathogenicity factors, the resistance profiles of *T. pyogenes* also vary depending on the animal species. Galán-Relaño et al. (2020) [11] showed that apramycin and oxytetracycline had higher minimal inhibition concentration (MIC)_90_ values when tested against isolates from cattle as opposed to isolates from sheep or goats in Spanish herds. Galán-Relaño et al. (2020) also found low MIC_90_ values for penicillin, amoxicillin, ceftiofur, enrofloxacin, and gentamicin. In fact, 93.7% of *T. pyogenes* isolates were susceptible to penicillin, and 77.2% were susceptible to erythromycin; additionally, 92.7% of isolates were resistant to sulfamethoxazole/trimethoprim [11].

Although the occurrence and various pathogenicity characteristics of *T. pyogenes* have already been studied, strain variation within different herds and environments has not been investigated. The aim of this study was therefore to obtain an overview of *T. pyogenes* appearance in herds to gain insights into its transmission behavior and to collect data for more targeted and deeper investigations.

## 2. Materials and Methods

### 2.1. Herds and Sampling

**Figure 2:**
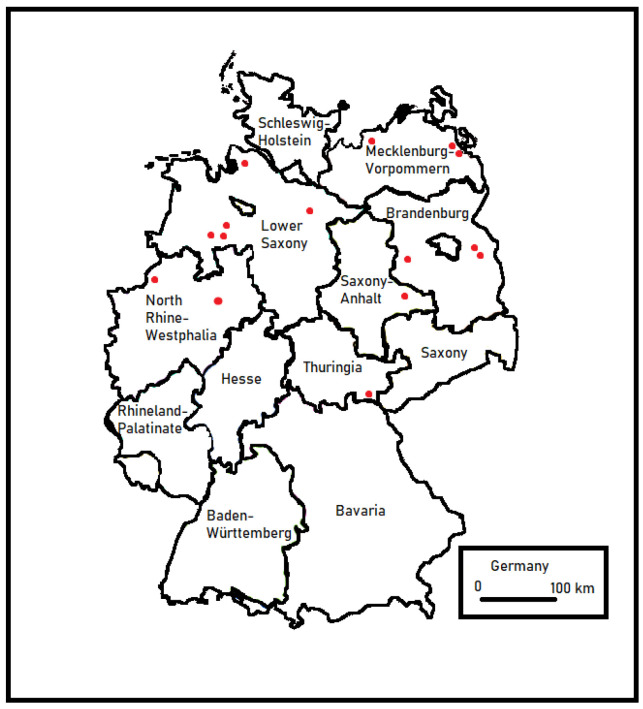
Locations of German farms involved in this study (red dots).

In this study, all clinical mastitis (CM) milk samples from German farms (Figure 2) that sent all their CM milk samples for microbiological diagnostics to the University of Applied Sciences and Arts (Hanover, Germany) were utilized. Therefore, *T. pyogenes* isolates were obtained from milk samples collected by veterinarians or farmers who had previously identified animals with clinical signs of mastitis. The sampling time frame was approximately one year for each farm. All farms with at least two *T. pyogenes* CM cases were included in this study. Quarters with single cases as well as those with recurrent *T. pyogenes* mastitis cases were considered.

To add isolates from subclinical *T. pyogenes* mastitis cases, all quarters of lactating cows from one farm were sampled every 14 days for a period of 140 days. This farm was located in Sweden and frequently visited as part of another study [12]. This offered the opportunity to obtain continuously taken samples from one entire dairy herd. All lactating cows of this herd were kept in a free-stall barn (separated into two groups based on lactation stage) and only had access to pasture during the summer. Throughout the sampling period (June–October), this herd had, on average, around 200 lactating dairy cows, a bulk tank SCC geometric mean of 195,000 cells/mL, and a CM incidence of 1.6 cases/100 cows per month within lactating and dry cows. Milk samples were taken in accordance with the guidelines of the German Veterinary Medical Association (GVA) [13] by disinfecting the teat ends with paper towels soaked in 70% ethanol. The first three milk streams were discarded, and the milk was milked into a sterile tube containing Ly-20 [14]. Additionally, gloves from the milkers (after each milking group (n = 2) once per farm visit) and flies from sticky fly traps (Redtop, 60 × 34.5 cm, poison free, Casa Verde GmbH, Dortmund, Germany) were sampled (four commercial sticky fly traps were hung up at one visit and remained until the next visit).

### 2.2. Milk Sample Analysis

Microbiological diagnostics were performed following the recommendations of GVA [13]. An amount of 10 μL of each sample was cultured on a quadrant of an esculin blood agar plate (Oxoid, Wesel, Germany) at 37 °C for 48 h. Isolates of *T. pyogenes* were identified as Gram-positive, catalase-negative, and esculin-negative rods (Figure 1a). The colonies showed a beta hemolysis around the colony (Figure 1b). A MALDI TOF analysis (Bruker Daltonics, microflex LT/SH smart, MBT Compass Library, V8) was performed to confirm the isolates as *T. pyogenes*. Also, isolates from mixed cultures were taken into account. All isolates were stored in Brain Heart Infusion Broth (BHI) (Merck, Darmstadt, Germany) supplemented with 20% anhydrous glycerol (Merck, Darmstadt, Germany) at −80 °C until further analysis.

Bacterial DNA was extracted using the DNeasy Blood and Tissue Kit (Qiagen Benelux B.V., Venlo, The Netherlands) in accordance with the manufacturer’s instructions. RAPD PCR was carried out in a 25 μL reaction volume containing 12.5 μL ReadyMix™ Taq PCR Reaction Mix (SigmaAldrich, Munich, Germany), 20 pmol of primer (Primer A, 5′-CTGGCGGCTTG-3′, according to Hijazin et al. 2013 [15]), 5 μL of the template, and water to make up the volume. Amplification was performed in an Mx3005 P qPCR system (Agilent, Santa Clara, CA, USA) using previously published methods, as described in Table 1. RAPD PCR products were stained with MIDORIGreen Direct (NIPPON Genetics Europe GmbH, Düren, Germany) and separated on 2% agarose gel. In addition, all products were tested side by side with identical samples to increase the accuracy of the results. Identical RAPD patterns of PCR products were defined as the same strain.

### 2.3. Fly and Glove Sample Analysis

All flies from one sampling date belonging to the same species were put together as one sample. This sample was analyzed twice to investigate the microorganisms from the outer and inner surfaces of the flies. First, the flies were shaken in one 2 mL reaction tube with 1 mL of sterile Ringer’s solution (Merck Kgaa, Darmstadt, Germany) for 10 s (Vortex Genie 2, lowest speed). All of the liquid was removed and utilized for microbiologic diagnostics. Then, the washed flies were filled again with 1 mL of half concentrated Ringer’s solution and homogenized with glass beads (Hybaid RiboLyser, 10 s Speed 4). The suspension was utilized for microbiological diagnostics. The liquids were aerobically cultured on Esculin Blood Agar (Oxoid, Wesel, Germany) for 48 h at 37 °C in serial dilution (−3 to −5). All of the hemolytic, catalase-positive colonies underwent a MALDI TOF analysis.

Each pair of gloves was fixed at the wrist to the opening of a Stomacher^®^ bag containing 100 mL of half concentrated Ringer’s solution to wash the microorganisms from the surface of the gloves by Stomacher mixing for 1 min. The liquid was aerobically cultured on Esculin Blood Agar for 48 h at 37 °C in serial dilution (−2 to −5). All of the hemolytic, catalase-positive colonies underwent a MALDI TOF analysis.

### 2.4. Contagiousness Index

To evaluate the contagiousness of *T. pyogenes* within each herd, an index termed the Contagiousness Index (CI) [16] was implemented. This index represents the number of isolates divided by the number of strains. If the number of isolates equals the number of strains, the CI is 1, indicating a low contagiousness of the *T. pyogenes* in a herd; on the other hand, an increasing CI develops by a decreasing diversity of the strains, which is represented by a growing number of isolates per strain, indicating a higher contagiousness of *T. pyogenes* within a farm.

## 3. Results

### 3.1. Total Numbers of T. pyogenes Isolates from Milk Samples

Over one year of sampling, 16 herds provided a minimum of three isolates from at least two cows infected with *T. pyogenes*. All in all, a total of 151 isolates from these farms were included in the study. A total of 41 out of 124 isolates (25%) were delivered by one herd (herd “H”) (Table 1).

### 3.2. Strain Variety within Herds and Contagiousness Index (CI)

In eight herds, all *T. pyogenes*-infected animals were infected by an individual strain (CI = 1). Subsequently, in the other eight herds, a minimum of two cows were infected with the same strain. Two herds (“H” and “J”) had three infected animals with an identical strain. In herd P, five animals had a subclinical infection with the same *T. pyogenes* strain. In this herd, the highest CI (CI = 1.8) was reached. All in all, four herds showed a *T. pyogenes* CI ≥ 1.5. In all herds, the CI never equaled the number of isolates per herd. The CI was not influenced by the herd size (Table 2).

In eight out of nine herds, multiple cow infections with the same strain were isolated from samples within a time frame of one month. Only in Farm “I”, two strains were detected approx. six months later in two other cows. In herd “J”, two cows tested positive for the same strain within one month, and seven months later, another cow was found infected with this strain. In herd “I”, one strain was found 4 months after its first detection in a second cow. In the same herd, another strain appeared 5 months after its first isolation in the sample from another cow. No strain matches could be found between any of the study herds.

### 3.3. Reinfections and Ongoing Infections

Reinfections of cows with a strain other than the previous one were detected in 9 herds out of 16 herds. Herd “H” delivered 25% of all *T. pyogenes* isolates and had the highest number of reinfections (5).

In six herds, ongoing infections (multiple samples from the same quarter of a cow containing the same strain) were found. Minimal time frames of approx. one week (herd “E” and “G”), one month (herd “K”), and two months (herd “A” and “H”) were identified for *T. pyogenes* infection durations. A long persistence was found in farm “O”; in this case, one strain was isolated again from the same cow after 7 months.

### 3.4. Quarter Level

All cows with *T. pyogenes* infection in multiple quarters at the same time harbored the same individual cow strain in all affected quarters, except for two cows in herd “H”. In detail, herd ”L” had two cows with, respectively, two affected quarters, and each of these cows had an individual *T. pyogenes* strain but the same strain within one cow. Herd “E” had a cow with all four quarters that was infected with one strain. In herd “P”, which only showed subclinical infections, the strain was not cow-specific. This means one cow was infected in three quarters, and one further cow was infected in two quarters with the same strain. In herd “H”, one cow was found infected with one strain in two quarters, and two cows were infected in two different quarters with all different strains in each quarter.

### 3.5. T. pyogenes Isolates from Environmental Samples

No isolates of *T. pyogenes* were found in the flies (36 fly traps were analyzed) or on the gloves (40 single gloves were analyzed) of the milkers.

### 3.6. Seasonality

In cooler times of the year, from October to April (7 months), 78 cows were identified as *T. pyogenes*-positive, and in the warmer period, from May to September (5 months), 44 cows had an *T. pyogenes* infection (Table 3).

For herd “P” (subclinical cases), no evaluation of seasonality could be made as the herd was sampled exclusively from June to October.

## 4. Discussion

All in all, *T. pyogenes* CM infections rarely appeared (Table 3) and showed a high strain variety (Table 2). In 50% of the study herds, no infections with matching *T. pyogenes* strains were found (CI = 1). Less than 25% of all study herds showed more than two cows infected with the same strain within one year. It should be noted that numerous farms, which systematically send their CM milk samples to our lab, were not included in this study because they did not provide enough *T. pyogenes* isolates. In all of the study herds, the CI never equaled the number of isolates per herd. Thus, the contagiousness of *T. pyogenes* appears to be quite low.

Nonetheless, in eight out of nine herds with multiple infections with matching strains, the infections with the same strain appeared in a timely manner within one month. This indicates some kind of contagious transmission behavior. Pathogens with special characteristics (e.g., adaptation to survive within the host and transmission during the milking process) may dominate at the herd level and therefore appear contagious [17,18]. Jayarao et al. (1993) [19] found less variety in strains from clinical mastitis than in those from subclinical mastitis. In herd P, which was sampled twice a month, only subclinical *T. pyogenes* infections were observed, and five cows (eight quarters) were infected with the same strain. A low variety of strains could likewise be caused by a contagious strain and/or by an environmental hotspot harboring a high concentration of the strain. However, if the subclinical cases had been investigated in all other study herds, a different overall picture might have emerged.

With regard to the infection process within a cow, we observed that all cows with *T. pyogenes* infection in multiple quarters at the same time harbored the same individual cow strain in the affected quarters. This indicates that *T. pyogenes* spreads easily between quarters of a cow.

In herd “J”, we detected the same strain in two different animals which were sampled seven months apart. This points towards the persistence of the strain within the herd. In addition, we also observed that a cow in herd “O” was found still infected with the same strain seven months after its first isolation from that cow, indicating a persistent infection within the quarter. However, the possibility of reinfection with the same strain cannot be excluded. Overall, we found several ongoing infections, and we might have missed more of them because we limited the sampling time frame to one year per herd. However, we did not collect control samples after the treatment of a *T. pyogenes* CM case, and no treatment protocols are available. Therefore, it remains unclear whether our observations show ongoing infections or reinfections. In a previous study [20], we found 22.2% of all *T. pyogenes* CM cases to be recurrent, of which half had been persistent. Due to the current study design, the observed numbers of isolates were highly influenced by farmers’ skills in detecting a CM and their willingness to collect milk samples from the detected cases. All in all, the farmers (especially those with a large herd size) send us the samples systematically, so compliance should not have a major impact on the result of the CI. We cannot exclude that some animals left the herd after a CM; therefore, the numbers of recurrent infections are possibly higher. In addition, the elimination of the affected animals could also influence the epidemiology within the herd.

Due to this study’s design, the herds were various. The size, treatment, milking, and housing conditions differed from farm to farm. Therefore, the farms were not directly comparable to each other, which is typical for dairy farming systems in Germany and many other regions. Nevertheless, in our study, we aimed to give an overview of *T. pyogenes* appearance in herds and possibly provide a direction for larger, more targeted studies.

As previously investigated [3,4], no overall seasonality in the appearance of *T. pyogenes* infections could be shown. Only subclinical cases in herd P appeared exclusively in the summer from June to August. In our study, herd P was located in Sweden, several hundred kilometers from the rest of the other study farms. It may be that the climate in Germany was generally milder than that in Sweden throughout the year. Summer mastitis might depend on multiple factors, e.g., heat stress or mixed infections. In our study, we did not consider other pathogens which were also found in *T. pyogenes* infections. It cannot be ruled out that in our study, other microorganisms are certainly partly or maybe even mainly responsible for the development of summer mastitis. However, we only included isolates from samples with a maximum of two different pathogens.

Due to our method, the strain diversity within one sample was not considered. Nonetheless, Oliver et al. [21] showed for other mastitis-causing pathogens that different colonies of a species from a single milk sample likely belong to the same strain.

The RAPD PCR method is frequently chosen for its suitability as a molecular diagnostic tool for a high throughput of isolates [17,21,22]. This method does have a propensity for false results since different amplified regions in different bacterial genomes may have the same length that would not be distinguishable from each other by RAPD PCR, and different species/strains may appear identical. However, the above results found more totally different amplification patterns than identical ones, suggesting that this bias should not have severely influenced the final conclusions.

Herd H delivered 25% of all isolates in this study. At first sight, a contagious *T. pyogenes* outbreak could have been considered. But after the RAPD PCR analysis, a high strain diversity with several reinfections, without a dominant strain, was apparent (CI = 1.1). A multifactorial event could have led to frequent *T. pyogenes* infections, e.g., many environmental spots harboring lots of *T. pyogenes* strains (e.g., wounds of cows), previous teat trauma, or poor immune statuses of cows and/or other microorganisms that have paved the way for *T. pyogenes* colonization.

We did not find *T. pyogenes* in any of the environmental samples in farm P. Perhaps there is another source of *T. pyogenes* in this farm which was not sampled during this study. We do not know whether the lack of detection was due to attributes of the dominating strain from subclinical *T. pyogenes* infection; maybe we would have a better chance of detection in farms with an increased incidence of clinical *T. pyogenes* mastitis and, therefore, increased shedding of *T. pyogenes*. Similarly, the effect of hygiene measures cannot be ruled out. It is also possible that *T. pyogenes* fell below the detection limit of our method for the environmental samples.

An extensive investigation of pathogen transmission is very complex, time-consuming, and costly. It was therefore necessary to look in advance at how this study can be set up on the basis of evidence. This pilot study had some limitations due to its design. As many dairy herds as possible should be included in order to obtain a far-reaching picture. We were therefore reliant on farmers to take samples. It is quite possible that not all clinical cases were recorded. Also, we do not know what happened to the affected animals because no treatment protocols, control samples, or culling data are available. Therefore, no clear conclusion can be made about the recurrence or persistence of *T. pyogenes* infections. The subclinical cases in the studied farms remained undetected and therefore uninvestigated except for one farm. Likewise, subclinical cases in farms which did not deliver enough clinical *T. pyogenes* mastitis cases to be included in this investigation remain unknown. No individual cow data (e.g., lactation and mastitis history) were collected; therefore, no risk factors for *T. pyogenes* infection on the cow level could be identified.

To achieve a high throughput in processing the isolates, RAPD PCR was chosen as the diagnostic method of choice. It provides information on whether the randomly generated DNA patterns of individual isolates match. So, in-depth genome-specific information could not be obtained in this study. For further investigations, next-generation sequencing (NGS) techniques should be used. By sequencing the genomes, NGS can visualize the distribution of *T. pyogenes* genomes within farms and provide reliable information about the transmission and likely source of *T. pyogenes.* Furthermore, an NGS-based investigation of milk microbiomes from affected cow quarters can deliver information about fastidious microorganisms which promote *T. pyogenes* infections. This information might help to find preventive measures against *T. pyogenes* mastitis and to break through the infection process, as there is no evidence-based *T. pyogenes* mastitis prevention method to date. With this study, we gained insights on where we can start with a more in-depth investigation to understand the infection pathways of *T. pyogenes*.

## 5. Conclusions

An extraordinarily frequent occurrence of *T. pyogenes* infections with clinical outcomes appeared in one single herd out of sixteen herds and was not attributable to a dominating strain. Instances of multiple cows being infected with the same *T. pyogenes* strain were rare and typically only involved a small number of animals at a time. But if several quarters of a cow were affected, it was very likely to be the same strain. Moreover, subclinical *T. pyogenes* infections harbored a dominant strain. Only one herd could be examined for subclinical *T. pyogenes* infections within this study. *T. pyogenes* infections from CM cases possibly persist and stay within a herd for at least 7 months. Furthermore, no seasonality of *T. pyogenes* appearance could be proven.

Future infection control must consider that *T. pyogenes* can persist in affected cows over a long period of time, that flies are not the sole reservoir of *T. pyogenes*, and that a high incidence of *T. pyogenes* cases is not necessarily due to contagious transmission. In addition, subclinical cases must not be ignored in infection control.

## Figures and Tables

**Table 1:** Strain diversity and occurrence.

Herd	No. of *T. pyogenes* Isolates	No. of Affected Cows	No. of Strains	No. of Cows Infected with the Same Strain ^1^	No. of Ongoing Infections ^3^ (Observed Duration ^4^)	No. of Reinfections (Duration until Reinfection)
A	11	10	9	2	1 (two months)	
B	3	3	3	0		
C	3	2	2	2		
D	3	3	2	2		
E	14	6	6	0	1 (one week)	2 (1 × one week, 1 × two weeks)
F	5	5	5	0		
G	5	4	3	2	1 (one week)	
H	41	31	28	3, 2, 2	1 (two months)	5 (2 × two weeks, 2 × one month, 1 × within four months)
I	5	5	3	2, 2		
J	10	10	8	3		1 (one month)
K	7	5	5	0	1 (one month)	1 (two months)
L	23	11	11	0		
M	3	3	3	0		
N	3	3	2	2		
O	9	8	8	0	1 (seven months)	
P ^2^	17	14	8	5, 2, 2		

^1^ The number of cows infected with the same strain. If several strains appeared in more than one cow, the number of positive animals per strain is given separated by a comma. ^2^ Exclusively subclinical *T. pyogenes* mastitis cases, ^3^ cases on a quarter level, ^4^ the time between two samples with an identical strain; no further samples were collected after one year of sampling.

**Table 2:** Contagiousness Index (CI).

Herd	Herd Size	No. of Affected Cows	No. of Strains	CI ^1^
A	998	10	9	1.1
B	345	3	3	1
C	728	2	2	1
D	924	3	2	1.5
E	556	6	6	1
F ^2^	1358	5	5	1
G	2448	4	3	1.3
H	1056	31	28	1.1
I	935	5	3	1.6
J	900	10	8	1.3
K	1217	5	5	1
L	1002	11	11	1
M	800	3	3	1
N	150	3	2	1.5
O	630	8	8	1
P ^2,3^	202	14	8	1.8

^1^ represents the number of isolates divided by the number of strains. ^2^ represents access to pasture. ^3^ represents subclinical infections only.

**Table 3:** Monthly distribution of *Trueperella pyogenes*-positive cows in dairy herds.

Herd	Month											
	JAN	FEB	MAR	APR	MAY	JUN	JUL	AUG	SEP	OCT	NOV	DEC
A			1		5	2	2	1				
B					1			1		1		
C					2							
D			1		2							
E		1	1	2		2	1				1	
F							2	2		1		
G	1								1	1	1	
H	6	1	4	3	1	1		2		4	5	7
I						2				2	1	
J			3	2	2						1	2
K		1	2			1	1		2			
L			1	1			2	1	1	4	3	3
M										2		1
N	1				1							
O		1	2	1		1		1	1		2	
P ^1^	n.s.^3^	n.s.	n.s.	n.s.	n.s.	3 (3) ^2^	8 (7) ^2^	3 (2) ^2^			n.s.	n.s.

^1^ Only subclinical mastitis cases sampled in the summer; ^2^ one ongoing infection; ^3^ n.s. = no sampling.

## Data Availability

Dataset available on request from the authors.
